# Ethical Data Release in Genome-Wide Association Studies in Developing Countries

**DOI:** 10.1371/journal.pmed.1000143

**Published:** 2009-11-24

**Authors:** Michael Parker, Susan J. Bull, Jantina de Vries, Tsiri Agbenyega, Ogobara K. Doumbo, Dominic P. Kwiatkowski

**Affiliations:** 1 Ethox Centre, University of Oxford, Oxford, United Kingdom; 2 Wellcome Trust Centre for Human Genetics, Oxford, United Kingdom; 3 Kwame Nkrumah University of Science and Technology, Kumasi, Ghana; 4 Malaria Research and Training Centre, University of Bamako, Bamako, Mali; 5 Wellcome Trust Sanger Institute, Hinxton, Cambridge, United Kingdom

## Abstract

Michael Parker and colleagues discuss the ethical issues associated with data release from genome-wide association studies in developing countries.

## The Challenge

Developing countries carry a disproportionate share of the global disease burden [1]. One main obstacle to developing better tools for disease prevention—such as vaccines against malaria, tuberculosis, and HIV—is our limited understanding of the underlying mechanisms of disease and protective immunity. Genome-wide association (GWA) studies provide a powerful way of getting at this problem by identifying genetic variants determining resistance or susceptibility to common diseases [2]–[4]. GWA studies to date have mostly focused on populations of rich countries, and there is a case for greater scientific investment in GWA studies relevant to the needs of developing countries.

GWA studies in developing countries raise a range of ethical issues. One aspect is the need to protect the rights of the individuals and communities who are the subjects of the research, e.g., by developing appropriate processes for valid consent [5]. Another aspect is to ensure that researchers and institutions in developing countries, who generate samples and data for GWA studies, are not put at a scientific disadvantage when they participate in the large collaborative networks that are needed to undertake this type of research [6]. We do not attempt to deal with here the full spectrum of ethical issues raised by GWA studies in developing countries, but focus specifically on the problem of releasing data to the broader scientific community.

There are strong scientific arguments for data release, as the full scientific value of a GWA study may not be realised unless it is analysed by different methods and combined with other datasets. For example, meta-analyses of GWA studies in different study populations have yielded many important discoveries not immediately apparent from individual studies. Several consortia undertaking large-scale GWA studies, such as the Wellcome Trust Case Control Consortium and the Genetic Association Information Network, have therefore adopted policies for releasing anonymised GWA data with appropriate regulatory procedures [4],[7]. The question we address here is how to develop policies and procedures for data release appropriate for GWA studies in developing countries.

Discussion about the role of data sharing in science is not new [8],[9]. Within the context of genomics, open access models of data release, which have their origins in the Bermuda Principles and the Fort Lauderdale agreement, have become common, and most large funding bodies now require the depositing of data in a centralised repository [10]–[12]. These moves reflect a belief that open access promotes the scientific use and social value of data.

While arguments for open access emphasise the ethical importance of promoting the availability of the results of genomic research to the scientific community and its potential to generate important public benefits [13]–[15], moves towards open access have also generated a significant literature concerning the compatibility of open access in genomic research with important ethical principles and values [15]. The range of ethical issues identified is extensive. It includes concerns about: privacy [16],[17], whether anonymity can be guaranteed [15],[17]–[19], security [17], the implications of collecting and storing vast amounts of data and about its uncertain future use [17], the implications of data release for populations [16],[18],[20] and for family members of participants [16],[17], the need to strike a proper balance between research and protection [15], the development of appropriate governance mechanisms [14],[15], the implications for trust, consent, and autonomy [16],[19],[21],[22], commercialisation [23], and the ethical importance of the sustainability of databases [24].

Despite this theoretical literature, there are no empirically grounded accounts of the ethical challenges in the development of data release policies in GWA studies in developing countries. Here we describe the development of a GWA data-release policy for the Malaria Genomic Epidemiology Network (MalariaGEN), a partnership of malaria researchers in over 20 countries supported by the Grand Challenges in Global Health initiative [25]. MalariaGEN investigators are using a range of genetic epidemiological approaches to investigate mechanisms of protective immunity against malaria, as part of the global effort to develop an effective malaria vaccine. MalariaGEN has sought to establish fair rules for sharing samples and data in large-scale research collaborations, a key principle being that contributing investigators retain ownership of the samples that they contribute to consortial projects [25]. Thus the datasets generated by individual investigators are not governed by the data-release policy described here, apart from specific items of phenotypic information that have been contributed by the investigators to consortial projects for the purpose of GWA analysis.

## Developing Policies and Mechanisms to Govern Release of GWA Data

**Figure 1:**
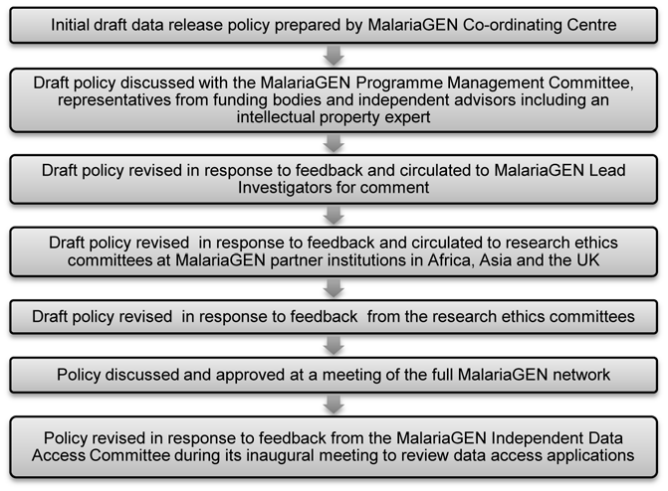
MalariaGEN's process for developing a GWA data-release policy.

Although MalariaGEN was founded with open access in mind [6], it was clear that the development of an effective, appropriate approach to GWA data release required widespread consultation across the network and with external stakeholders (see Figure 1). In what follows, we outline some of the key issues arising during this process and how these were addressed.

### From Open Access to Managed Open Access

The Fort Lauderdale agreement calls for the immediate release of genomic data to the scientific community, constrained only by the need to protect the rights of data producers to pursue their stated scientific aims without being “scooped” by those who gain access to their data [26]. Although MalariaGEN investigators supported this general principle, after extensive discussion and consultation it was concluded that it would be inappropriate to provide entirely open public access to GWA data on individuals accompanied by specific phenotypic data.

One factor in this decision was the scientific importance of information about an individual's ethnic group. Many communities in Africa have considerable complexity in population structure, i.e., they are composed of several ethnic groups that differ in their frequency of common genetic variants, so that knowledge about an individual's ethnic group is needed by researchers to determine whether an apparent genetic association is truly related to disease susceptibility or is an artefact caused by these ethnic differences. In theory, samples from different ethnic groups or geographic regions might be distinguished without naming them explicitly, e.g., by labelling them 001, 002, etc., but a relatively simple statistical calculation would break such a code.

**Figure 2:**
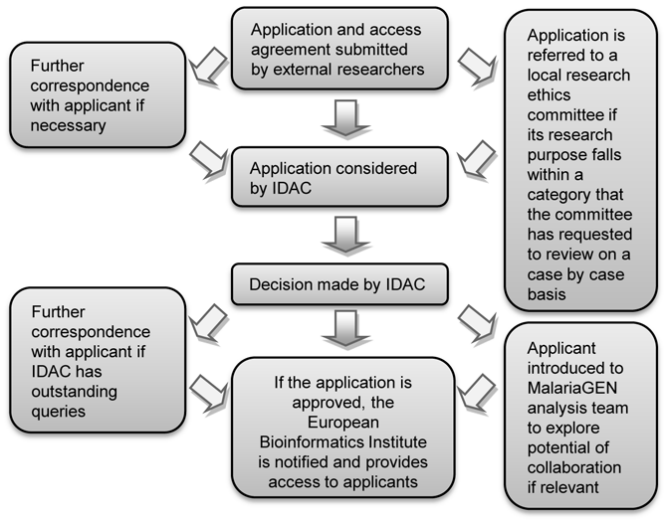
Data application process.

Following consultation, it was therefore agreed that access to MalariaGEN datasets would be mediated via an independent data-access committee (IDAC) (see Figure 2), and that researchers would be granted access to genotyping data and to a limited amount of clinical and demographic data only after signing a legally binding data-access agreement (see http://www.malariagen.net/resource/2).

MalariaGEN took some time to reach a consensus about the IDAC's composition, role, and remit. Questions arising during the process included how best to strike an appropriate balance between the independence of the DAC and ensuring sufficient expertise to review applications, and how to ensure resources for its long-term sustainability. Stakeholders consulted during development of the policy emphasised that the IDAC would need to be able to take into account the interests of research participants, communities, ethics committees, and MalariaGEN Principal Investigators from developing countries. In the context of a collaborative network involving research groups in many countries, it was not feasible to have representatives for each sample set on the IDAC. Given this, the decision was made to appoint a small number of members (six in the first instance) each with multiple relevant areas of expertise, to facilitate timely and rigorous review. To complement the IDAC, it is proposed to establish a broader consultative group involving partner institutions, ethical review bodies, and funding agencies that will receive regular reports of the uses made of the released data and may be asked to consider issues of policy from time to time by IDAC. This mechanism will enable widespread engagement with the process of data release, without requiring members to undertake case-by-case consideration of all data access applications.

### Acceptable Uses of Data

A further issue arising in consultation concerned the restrictions to be placed upon the kinds of research allowable using data. For example, should the use of data be restricted to “medical research” or should anthropological research be permitted? Clearly, the ethical release and use of data requires respect to be paid to the conditions under which the original consent was obtained. While there is currently a lively international debate about appropriate models of consent for GWA studies [5],[17],[27], many MalariaGEN samples were collected at a time when current developments in genomic science and data sharing were unenvisaged. The potential uses and benefits of data often extend far beyond the original purpose specified in the consent form. There are arguments in favour of the use of such data, given appropriate safeguards and where the use might reasonably be considered to be something to which the donor would have consented. But, if it is not realistic to go back to participants to obtain their consent, how should decisions about appropriate access and research use be made?

The IDAC came to the view that the need to interpret the scope of the original ethics approval and consent meant that determining acceptable forms of research on data would require input from relevant research ethics committees. At present IDAC engages with local ethics committees for specific sample sets as they become due for release, to determine exactly how broad a range of research purposes is considered acceptable. In some cases ethics committees may reserve the right to consider applications for access for what they consider to be borderline research purposes on a case-by-case basis (see http://www.malariagen.net/resource/2).

### Timing

The Fort Lauderdale agreement emphasises that the scientific work and aspirations of data producers should be recognised and not undermined by open access. In the context of genomic data produced by researchers in developing countries, there is a possibility that were such data released immediately to the wider scientific community, these researchers would be “scooped” by those from richer countries. This suggests a level of protection might be appropriate.

The primary purpose of open access is the promotion of appropriate research. MalariaGEN takes the view that capacity-building in developing country research is important both to the future success of addressing the health care needs of developing countries through the development of local expertise, and to promoting the trust underpinning the viability of multinational scientific networks upon which such success to a large degree depends. The Network came to the view that its data-sharing policy must, in addition to promoting science in the short term, promote science and the conditions necessary for science relevant to developing countries in the longer term.

For these reasons, the policy allows for a delay in data release for up to nine months after MalariaGEN investigators at the study site have access to their dataset. This, combined with other capacity-building measures, should assist in balancing the significant differences in analytic capacity present in developed and developing countries. Where principal investigators from the study site agree, data may nevertheless be released immediately along with notification of areas of research the MalariaGEN Network and individual principal investigators are undertaking with the dataset (see, for example http://www.malariagen.net/resource/2). Applicants accessing the data are asked to respect these areas of research and refrain from publishing analyses in them prior to the initial MalariaGEN publications on those topics.

## Looking Forward

The purpose of the MalariaGEN data-release policy is to promote the scientific use and the social value of its data. There is a need to find effective mechanisms to communicate the key findings of the research, and how the released data have been used by the scientific community, to participating communities and to local research ethics committees. There is also a need to find effective and appropriate ways of conveying the purpose of the research and exploring its social and cultural implications if local communities are to be able to participate effectively in debates about the release of GWA data. This need is of particular importance in relation to data on ethnicity, and how individual ethnic groups are to be identified and labelled. Genetic researchers need to work with social scientists and with research ethics committees to understand how such issues are perceived by local communities, and to ensure that these views are respected in the released data.

It is our view that an ethical data-release policy must, in addition to providing adequate protections for research participants and their communities, be combined with adequate protections for the research aspirations of developing country scientists and with capacity-building activities to ensure that those aspirations have the potential to be realised. Collaborative global health research capable of addressing the needs of people in developing countries depends crucially upon the building of capacity in developing country sites to enable locally held clinical and phenotypic data to be analysed locally in combination with genotype data. This has the potential to lead to the identification of key site-specific factors that play a role in the development of malaria. What this means is that the sharing of genotypic and phenotypic data is by itself insufficient without the sharing of expertise. For this reason MalariaGEN is complementing its data-release policy with a programme for the training and support of data-fellows in malaria-endemic partner sites to strengthen capacity for genetic data analysis across the network. Most data-fellows work within the team of a MalariaGEN investigator and have responsibilities for managing the team's data. Senior data-fellows help to provide mentorship for the group (http://www.malariagen.net/resource/2).

## Abbreviations

GWA: genome-wide association
IDAC: independent data-access committee
